# Utility of speckle tracking echocardiography in measuring systemic right ventricular systolic function for patients with d-transposition of the great arteries status post atrial switch procedure: a comparison with cardiac magnetic resonance imaging

**DOI:** 10.1186/1532-429X-18-S1-P176

**Published:** 2016-01-27

**Authors:** Conor Masterson, David Saudek, Scott Cohen, Julie Slicker, Aaron Kinney, Mary Krolikowski, Margaret M Samyn

**Affiliations:** grid.414086.f000000010568442XMedical College of Wisconsin/ Children's Hospital of Wisconsin, Milwaukee, WI USA

## Background

Patients with d-transposition of the great arteries (D-TGA), status post atrial switch, are at risk for developing systemic right ventricular (RV) dysfunction. Echocardiographic (Echo) assessment of RV function is subjective because complex RV geometry does not allow accurate determination of ejection fraction (EF). RVEF measured by cardiac magnetic resonance imaging (MRI) is the gold standard for quantitative assessment of systemic RV function. New Echo measures of ventricular deformation allow for quantitative assessment of RV function. The primary aim was to explore the correlation of global peak longitudinal strain (GPLS) of the systemic RV with MRI RVEF for patients with D-TGA status post atrial switch. The secondary aim was to characterize MRI and clinical findings in this population.

## Methods

This was a retrospective review of medical records for D-TGA patients with atrial switch followed at Children's Hospital of Wisconsin from 1960 to 2015. All patients who underwent Echo within 1 year of cardiac MRI, exercise test, and clinic visit were included. Echo derived GPLS was determined by tracing systemic RV endocardial border from apical 4-chamber view using Tomtec^®^ software. MRI assessment of RV mass and volume was made from endocardial and epicardial tracing performed on contiguous short axis images. Medians and ranges were calculated for continuous variables, while frequencies and percentages for categorical variables. Pearson's correlations were calculated between 2 continuous variables. Student t-test was used to assess significant difference between GPLS stratified by MRI EF above and below 45%.

## Results

Twenty-seven (16 males) patients were enrolled. The median age was 30 years (range 19-50 yrs) at time of MRI. Median age at atrial switch was 10.8 months (range 0.01-10.50 yrs). Seventy-four percent were NYHA class I and 26% class II. There was no significant correlation between GPLS and MRI RVEF (p = 0.844). When comparing GPLS in patients with MRI RVEF >45% (n = 20) and <45% (n = 7), GPLS was similar. MRI RVEF correlated weakly with Echo RVEF (r^2^ = 0.258, p = 0.007). MRI RVEF correlated positively with RV mass index when the interventricular septum (IVS) was attributed to the RV (r^2^ = 0.246, p = 0.008) and with RV EDV index (r^2^ = 0.391, p = 0.001). Neither GPLS nor any MRI parameter correlated with exercise endurance (median = 8.97 minutes, range 3.65 - 12.58 min) or NYHA class.

## Conclusions

In a small cohort of adult patients with D-TGA status post atrial switch, GPLS of the systemic RV did not correlate with MRI RVEF or exercise endurance, nor was there a difference in GPLS when patients were stratified by MRI RVEF. MRI RVEF, though, correlated with RV EDV index and RV mass index (IVS to RV), but not with exercise endurance. These findings suggest that Echo derived GPLS is not a useful surrogate of MRI RVEF. A larger, multi-center investigation of patients with atrial switch may illuminate MRI's role in assessing the long-term clinical implications of the systemic RV.

